# Role of Temperature and Coinfection in Mediating Pathogen Life-History Traits

**DOI:** 10.3389/fpls.2018.01670

**Published:** 2018-11-20

**Authors:** Elise Vaumourin, Anna-Liisa Laine

**Affiliations:** Research Centre for Ecological Change, University of Helsinki, Helsinki, Finland

**Keywords:** abiotic interactions, biotic interactions, life-history evolution, overwintering success, pathogen evolution, *Plantago lanceolata*, *Podosphaera plantaginis*

## Abstract

Understanding processes maintaining variation in pathogen life-history traits is a key challenge in disease biology, and of importance for predicting when and where risks of disease emergence are highest. Pathogens are expected to encounter tremendous levels of variation in their environment – both abiotic and biotic – and this variation may promote maintenance of variation in pathogen populations through space and time. Here, we measure life-history traits of an obligate fungal pathogen at both asexual and sexual stages under both single infection and coinfection along a temperature gradient. We find that temperature had a significant effect on all measured life-history traits while coinfection only had a significant effect on the number of sexual resting structures produced. The effect of temperature on life-history traits was both direct as well as mediated through a genotype-by-temperature interaction. We conclude that pathogen life-history traits vary in their sensitivity to abiotic and biotic variation in the environment.

## Introduction

Pathogens are a major threat to human health and food security (Strange and Scott, 2005; Jones et al., 2008). The success of pathogens may be attributed to their evolutionary potential which allows them to emerge, adapt, and persist in space and time within their host populations (Mundt, 2002; Friesen et al., 2006). Hence, understanding processes maintaining variation in pathogen life-history traits is a key question in evolutionary ecology and a major challenge for the design of disease control programs (Galvani, 2003; Grenfell et al., 2004). Environmental variation may be a powerful process maintaining variation in pathogen populations if infection outcomes depend on conditions directly or through genotype-by-environment interactions, and if variation is spatially and/or temporally structured (e.g., heterogeneous landscapes and seasonality). To understand the role of environmental variation in maintaining variation in pathogen populations, to date most studies have focused on the abiotic environment (e.g., climatic conditions, Harvell et al., 2002). Recently it has become evident that pathogens often occur simultaneously with other strains or species of pathogens colonizing the same host (Tollenaere et al., 2016). Thus, biotic variation in the co-occurring pathogen community may be an overlooked component of the processes maintaining variation in pathogen populations.

The abiotic environment has been shown to affect the evolutionary trajectories of pathogen life-histories (Blanford et al., 2003; Fels and Kaltz, 2006). Variation in temperature represents one of the most ubiquitous sources of environmental variation, and is known to greatly affect biochemical, physiological and behavioral processes of organisms. Pathogens with a free transmission stage are considered particularly vulnerable to variation in temperature (Truscott and Gilligan, 2003). In many pathosystems, temperature has been shown to affect pathogen ability to establish or maintain infection, its latency as well as its aggressiveness (e.g., Burdon, 1987; Thomas and Blanford, 2003; Fels and Kaltz, 2006). There is increasing evidence that the effect of temperature on pathogen fitness may be mediated through genotype-by-environment (G × E) interactions, suggesting that adaptation to biotic and abiotic habitats may be strongly linked (Ferguson and Read, 2002; Thomas and Blanford, 2003; Price et al., 2004; Mitchell et al., 2005; Fels and Kaltz, 2006). For example, it has been shown that the diversity *Arcobacter* populations is modulated by a temperature gradient (Fisher et al., 2014).

More recently, in addition to the surrounding abiotic environment, the biotic environment has been also highlighted to impact the maintenance of the variation in pathogen populations. For example, epidemic outcomes have been shown to change when multiple pathogens are present on a same host (Susi et al., 2015a). Strains under coinfection are generally expected to compete for the same limited resources provided by the host. If this process was acting alone, it could favor higher host exploitation rates (i.e., higher virulence) under coinfection than under single infection (Alizon, 2013). For many pathogens coinfection is also the pre-requisite for sexual reproduction, and thus changes in pathogen growth rates under coinfection may also represent facilitation to increase the probability of generating of new allelic combinations between the coinfecting strains (Otto, 2009; Carter et al., 2013). Thus, coinfection may largely contribute to maintain the genetic variation in pathogen life-history traits and can speed up the adaptation to environmental change (Becks and Agrawal, 2012).

Both abiotic and biotic environments may have strong impacts on infection outcomes, but little is known about their relative importance, and whether outcomes of coinfection are mediated by temperature (but see Marçais et al., 2017). In this study, we investigate how pathogen life-history traits that constitute both asexual and sexual stages are affected by the biotic environment (i.e., coinfection) and abiotic environment (i.e., temperature). Despite its ecological importance, the sexual stage is difficult to study for many pathosystems. Our study is focused on the interaction between *Podosphaera plantaginis* – *Plantago lanceolata* (powdery mildew – ribwort plantain). Given that this pathogen completes its entire life-cycle on the surface of the host plant, we are able to monitor the life-history traits visually in a non-destructive manner. Another advantage of this pathosystem is that *P. plantaginis* is a homothallic fungi (i.e., self-fertile, Tollenaere and Laine, 2013) so the production of the sexual structures (i.e., chasmothecia) can be achieved under both single strain infections as well as in coinfections. Thus, we can evaluate the impact of coinfection and temperature on this critical life-history trait affecting overwinter survival.

## Materials and Methods

### The Pathosystem: *Podosphaera plantaginis – Plantago lanceolata*

We focused our study on the powdery mildew, *Podosphaera plantaginis* (*Erysiphales*, Ascomycota), which is an obligate fungal pathogen naturally infecting host plant *Plantago lanceolata*, the ribwort plantain. The epidemic cycle of the powdery mildew starts with the germination of a spore on a susceptible host resulting in lesion where clonal spores which are wind-dispersed are produced. To survive the winter, resting structures (chasmothecia) are produced. Each resting structure contains eight sexually generated ascospores, which initiate new infections in the spring upon their release. In *P. plantaginis* chasmothecia production can be achieved via haploid selfing of pure strains as well as outcrossing between two different strains infecting the same host plant (Tollenaere and Laine, 2013).

The dynamics of *P. plantaginis* in its fragmented host population network have been intensively studied in the Åland Islands (50 km × 70 km area) in southwest Finland since 2001 (Ojanen et al., 2013). The study system consists of approximately 4 000 host populations that are surveyed annually for the presence of *P. plantaginis*. The powdery mildew infection is visually conspicuous as whitish mycelia on the leaves. Previous studies have shown that prevalence of the pathogen is low in this system, with 2–17% of the host populations being infected each year in the metapopulation. *Podosphaera plantaginis* persists as highly dynamic metapopulation with frequent extinctions and (re)colonizations (Laine and Hanski, 2006; Jousimo et al., 2014). Genotyping has revealed considerable genetic diversity in this pathogen metapopulation, with most of the strains found in only one or few localities with a small subset of strains being common across multiple host populations (Tollenaere et al., 2012). The three strains chosen for our experiment – S1 (genotype i1), S2 (genotype 4000) and S3 (genotype 876-1) – are temporally and spatially widely distributed in the Åland metapopulation (Supplementary Figure 1).

Prior to the experiment, we carried out repeated cycles of inoculations to obtain adequate stocks of sporulating fungal material for the inoculation trials. The inoculations were done on petri dishes on detached host leaves of a *P. lanceolata* genotype (*490-15*) that has been characterized as broadly susceptible during previous experimental and maintenance work. For the experiment we used conidial chains from lesions that were 16–18 days old. To have sufficient leaf material for the experiment, we produced multiple clones by placing mother plants growing in pots with a perforated bottom in pots filled with vermiculite. After 9 weeks the mother plant was cut from the roots it had developed in the pot of vermiculite. Roots in the vermiculite yielded new rosettes, which were planted into pots with sand-rich humus. The plants were grown under glasshouse conditions, with 16 h of light and a temperature of +22°C.

### Measuring Asexual and Sexual Pathogen Life-History Traits Under Single Versus Coinfection Along a Temperature Gradient

In order to investigate how pathogen life-history traits are impacted by the biotic environment (i.e., coinfection) and by the abiotic environment (i.e., temperature), we conducted an inoculation experiment where we recorded life-history traits of strains on their own (single infection) or with another strain (coinfection). We measured pathogen life-history traits under three different temperatures, +17°C, +20°C and +23°C. This range represent typical growing season temperatures that *P. plantaginis* experiences in the Åland archipelago (Laine, 2008). We measured two life-history traits that constitute the asexual stage (i.e., affecting pathogen growth): time from inoculation to germination and time from germination to sporulation. We also quantified three life-history traits of the sexual stage (i.e., affecting pathogen overwinter survival): time from sporulation to mature chasmothecia, total number of chasmothecia and proportion of viable chasmothecia. Given that this pathogen completes its entire life-cycle on the surface of the host plant, we were able to monitor life-history traits visually in a non-destructive manner. All inoculations were performed on the broadly susceptible host genotype (*490-15*).

To provide single vs. coinfection comparisons under different temperature conditions, each of the three strains was inoculated both individually as well as with another strain in a fully crossed design under the three different temperatures (+17°C, +20°C and +23°C). A cross-inoculation of a strain with itself is considered as a single infection and a cross-inoculation of a strain with another strain is considered as a coinfection. *Plantago lanceolata* leaves were placed on a moist filter paper in a Ø 9 cm Petri dish. Three conidial spore chains of both strains (i.e., six conidial chains at each inoculation site) were carefully placed on the same spot of a *P. lanceolata* leaf by single hair inoculation technique (Tollenaere and Laine, 2013). On each leaf we inoculated three distinct spots resulting in 54 sites of inoculation for each strain pairing per temperature. All inoculated leaves inside Petri dishes were kept at their respective temperatures with a 16L/8D photoperiod in growth chambers. We included non-inoculated leaves as negative controls. All *P. lanceolata* leaves used in the experiment were harvested from same-aged clones that were maintained in the greenhouse.

For each inoculated site, using a dissecting microscope, we measured daily the life-history traits related to the pathogen growth. Then, 14 days after the appearance of the first mature chasmothecia, the number of total chasmothecia produced were counted under a dissecting microscope and the viability of approximately 10 mature randomly collected chasmothecia were measured. To measure their viability, we have used the vital stain fluorescein diacetate (FDA) method (Firstencel et al., 1990; Ficke et al., 2002; Vági et al., 2016). This method consists of gently crushing chasmothecia on microscope slides in a droplet of a solution containing FDA (10 μg/mL). Slides were then scored for fluorescing ascospores under an epifluorescence microscope. FDA is lipophilic, membrane-permeable, non-fluorescent and it is hydrolyzed onto polar fluorescent molecules in the cytoplasm of living cells. These molecules remain in the cytoplasm of viable ascospores with intact cell membranes and exhibit intense green fluorescence when examined under blue light (465–495 nm) thereby signaling chasmothecia viability. A chasmothecium was scored as being viable if it contained any ascospores that exhibited intense green fluorescence.

### Statistical Analyses

To understand how temperature and coinfection affected variation in the asexual life-history traits - time from inoculation to germination, time from germination to sporulation and time from sporulation to mature chasmothecia – we used survival models as implemented in R-package survival (Therneau, 2015) with Cox Proportional Hazards model (Cox, 1972). All survival models had temperature, first strain identity, coinfection and their respective interactions with the temperature as fixed explanatory effects. The identity of the second coinfecting strain and the leaf were considered as random factors.

To understand how temperature and coinfection affected variation in the sexual life-history traits – total number of chasmothecia and proportion of viable produced chasmothecia – we fitted generalized linear mixed models. For the model estimating total number of produced chasmothecia we defined a normal error structure and an identity link function, and for the model analyzing proportion of viable chasmothecia we defined a binomial error structure and a logit link function. Then for each model, the temperature, first strain identity, coinfection and their respective interactions with the temperature were fixed explanatory effects and the second coinfecting strain and leaf were considered as random factors.

## Results

**FIGURE 1:**
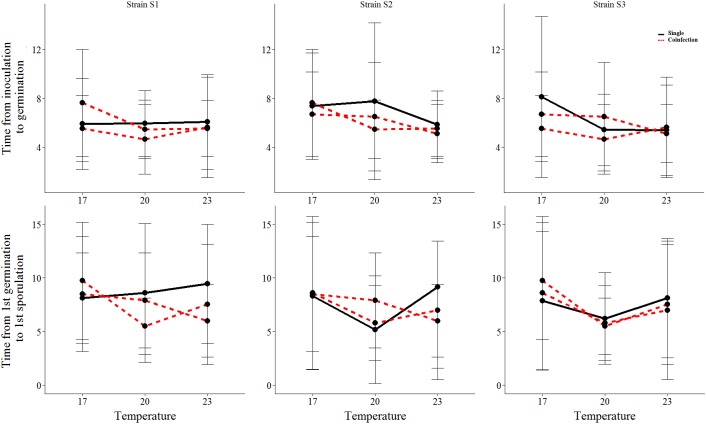
The mean and standard deviation of each asexual life-history trait of the three *Podosphaera plantaginis* strains in single vs. coinfection along a temperature gradient. Single infections are depicted by black lines and coinfection by red lines. The time is indicated in days.

**FIGURE 2:**
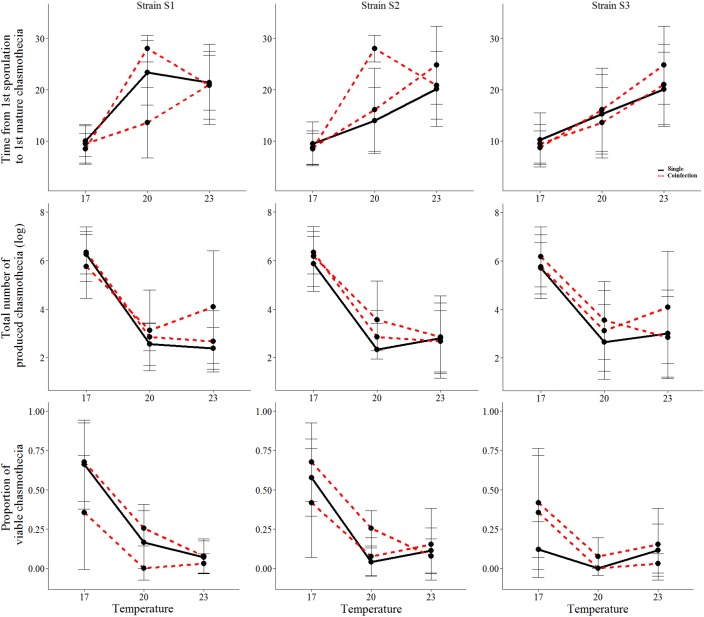
The mean and standard deviation of the sexual life-history traits of the three *Podosphaera plantaginis* strains in single vs. coinfection along a temperature gradient. Single infections are depicted by black lines and coinfection by red lines. The time is indicated in days.

We found that all measured life-history traits are significantly affected by temperature (Figures 1, 2 and Table 1). The average timing of each life-history event at each temperature and strain is summarized in Table 2 and in Supplementary Figure 2. At the lowest temperature, +17°C, we find all sexual life-history traits to perform better than at higher temperatures (Table 2). For example, for strain S1 the time from first sporulation to first mature chasmothecia is 2.1 times faster at +17°C than at +23°C, 2.1 times faster for strain S2 and 2.0 times faster for the strain S3. The total number of produced chasmothecia is 73.3 times higher at +17°C than at +23°C for strain S1, 13.8 times higher for strain S2, and 10.0 times higher for strain S3. The proportion of viable chasmothecia is 9.4 times higher at +17°C than at 23°C for the strain S1, 5.3 times higher for the strain S2 and the same for the strain S3. For the asexual life-history traits studied – time from inoculation to germination and time form germination to sporulation – the effect of temperature tended to be the opposite. Strains were typically faster to germinate and launch spore production at +20°C and +23°C than at +17°C (Table 2 and Figure 1).

**Table 1:** Results of the GLMMs analyzing infection development of *Podosphaera plantaginis* measured under single vs. coinfection under a temperature gradient.

*Source*	χ*^2^*	*P*
**Time from inoculation to germination**		
First strain identity	1.20	0.548
Coinfection	2.99	0.084
Temperature	14.57	**<10^−2^**
First strain identity × Temperature	7.59	0.108
Temperature × Coinfection	0.31	0.858
**Time from first germination to first sporulation**		
First strain identity	2.83	0.243
Coinfection	1.26	0.261
Temperature	13.31	**<10^−2^**
First strain identity × Temperature	1.41	0.842
Temperature × Coinfection	4.08	0.130
**Time from first sporulation to first mature chasmothecia**		
First strain identity	8.51	**0.014**
Coinfection	0.51	0.475
Temperature	149.41	**<10^−2^**
First strain identity × Temperature	17.70	**<10^−2^**
Temperature × Coinfection	2.85	0.241
**Total number of produced chasmothecia**		
First strain identity	2.08	0.353
Coinfection	6.87	**<10^−2^**
Temperature	386.85	**<10^−2^**
First strain identity × Temperature	12.72	**0.013**
Temperature × Coinfection	1.70	0.428
**Proportion of viable chasmothecia**		
First strain identity	57.06	**<10^−2^**
Coinfection	1.00	0.317
Temperature	135.64	**<10^−2^**
First strain identity × Temperature	25.77	**<10^−2^**
Temperature × Coinfection	0.39	0.822

*Statistically significant results are shown in bold.*

**Table 2:** The means and standard deviations of the measured life-history traits for each temperature and strain under single infection.

		+17°C			+20°C			+23°C		
		S1	S2	S3	S1	S2	S3	S1	S2	S3
Time from inoculation to germination	Mean	**5.90**	7.37	8.12	5.94	7.76	**5.42**	6.07	5.85	**5.39**
	*SD*	*3.71*	*4.34*	*6.55*	*2.70*	*6.41*	*2.93*	*3.87*	*2.76*	*3.69*
Time from 1st germination to 1st sporulation	Mean	8.14	8.33	**7.88**	8.59	**5.18**	6.23	9.45	9.15	**8.12**
	*SD*	*4.21*	*6.84*	*6.46*	*6.47*	*5.03*	*4.27*	*5.55*	*7.54*	*5.56*
Time from 1st sporulation to 1st mature chasmo	Mean	10.00	**9.47**	10.24	23.33	**14.00**	15.23	21.33	20.13	**20.07**
	*SD*	*2.98*	*4.24*	*5.26*	*6.28*	*6.44*	*7.76*	*5.33*	*7.29*	*7.25*
Total number of produced chasmo	Mean	**824.19**	546.43	503.12	16.78	**25.00**	18.62	11.25	39.67	**50.33**
	*SD*	*719.76*	*432.12*	*648.03*	*11.40*	*36.73*	*27.52*	*13.29*	*74.88*	*65.66*
Proportion of viable chasmo	Mean	**0.66**	0.58	0.12	**0.17**	0.04	0.00	0.07	0.11	**0.12**
	*SD*	*0.28*	*0.25*	*0.18*	*0.24*	*0.09*	*0.00*	*0.10*	*0.14*	*0.17*

*The time is indicated in days. The best performing strain at each temperature is indicated in bold.*

Coinfection did not have a significant effect on the asexual life history traits measured. However, we found that under coinfection significantly more chasmothecia are produced (Figure 2 and Tables 1, 3). For the strain S1 2.0% chasmothecia are produced more under coinfection, 45.6% more for the strain S2 and 63.4% more for the strain S3. The proportion of viable chasmothecia was not significantly different between single infection and coinfection (Figure 2 and Table 1). The interaction between temperature and coinfection was not significant for any of the measured life-history traits (Table 1).

**Table 3:** The means and standard deviations of the measured life-history for each temperature and strain under coinfection.

		+17°C			+20°C			+23°C		
		S1	S2	S3	S1	S2	S3	S1	S2	S3
Time from inoculation to germination	Mean	6.37	7.06	**6.13**	**4.98**	6.07	5.53	5.59	**5.30**	5.36
	*SD*	*3.58*	*3.84*	*3.14*	*2.68*	*3.68*	*3.74*	*3.33*	*2.32*	*3.29*
Time from 1st germination to 1st sporulation	Mean	9.25	**8.56**	9.16	6.46	6.69	**5.65**	6.81	**6.55**	7.26
	*SD*	*5.38*	*6.43*	*6.33*	*3.59*	*3.98*	*3.02*	*4.70*	*5.26*	*6.01*
Time from 1st sporulation to 1st mature chasmo	Mean	9.08	**8.62**	9.07	20.00	19.50	**15.27**	**20.92**	23.00	23.34
	*SD*	*3.45*	*3.12*	*3.51*	*8.88*	*8.65*	*7.40*	*6.91*	*7.25*	*7.68*
Total number of produced chasmo	Mean	765.22	**804.16**	794.07	17.56	**49.86**	46.67	86.42	35.67	**93.79**
	*SD*	*1211.90*	*784.58*	*1221.78*	*23.27*	*57.86*	*59.14*	*192.60*	*59.00*	*186.34*
Proportion of viable chasmo	Mean	0.48	**0.52**	0.39	**0.17**	0.13	0.06	0.06	**0.12**	0.11
	*SD*	*0.36*	*0.33*	*0.35*	*0.15*	*0.14*	*0.11*	*0.09*	*0.18*	*0.19*

*The time is indicated in days. The best performing strain at each temperature is indicated in bold.*

We found significant variation among strains in the timing of chasmothecia production and their viability (Figure 2 and Table 1). We also found that for all traits measured, no strain can outperform all others in all temperatures (Figures 1, 2, Table 2, and Supplementary Figure 2). This trend was most pronounced when measuring the timing and total number of chasmothecia, as well as proportion of viable chasmothecia, as evidenced by the statistically significant strain identity × temperature interaction (Figure 2 and Table 1).

## Discussion

Understanding processes maintaining variation in pathogen life-history traits is a long-standing challenge in disease ecology as this variation underlies risks of pathogen emergence and risks of infection (Galvani, 2003; Grenfell et al., 2004). Here, we have measured life-history traits at both asexual and sexual stages of an obligate fungal pathogen. We find that when we simultaneously investigate the effect of abiotic and biotic environmental variation, only temperature had a consistent and significant impact on all measured pathogen life-history. For several of the measured traits, the pathogen strains also differed significantly in how they responded to variation in temperature. Coinfection had a significant direct effect on the number of produced chasmothecia. Our finding suggests that abiotic variation may be more important in maintaining variation in pathogen populations than biotic variation.

In line with a previous study (Laine, 2007), our results demonstrate that the asexual life-history traits leading to infection are significantly affected by temperature. For the asexual life-history traits the optimal temperature depended on the life-history trait as well as strain identity. In general, although the specific effects of temperature vary among pathosystems, it has been shown that temperature may impact all key pathogen life-history traits including the ability to establish infection, subsequent development as well as transmission (Burdon, 1987; Thomas and Blanford, 2003; Fels and Kaltz, 2006). Here, we also find that temperature significantly affects traits linked with the sexual stage. Our inoculation study revealed that at +17°C, the number and viability of produced chasmothecia was the highest for all three strains. This effect of the temperature is in line with the biology of the powdery mildew, as the production of the sexually produced resting structures may be partly triggered by lower temperatures toward the end of the growing season (Tollenaere and Laine, 2013).

Genotype-by-environment interactions are considered an important mechanisms maintaining variation. In support of this, here we find that for nearly all measured life-history traits, the strains switch ranks along the temperature gradient. Hence, no strain outperforms all others across all temperatures. This trend was statistically significant when measuring all traits linked with sexual reproduction: the timing and number of produced chasmothecia, as well as their viability. These represent core fitness traits in *P. plantaginis*, as chasmothecia production is strongly linked with the ability to survive over winter (Tack and Laine, 2014). Although the experiment only included three strains of *P. plantaginis*, it is in line with previous work demonstrating significant temperature and genotype x temperature effects on asexual life-history traits using a diverse range of *P. plantaginis* strains (Laine, 2004, 2008). An interesting avenue of future work would be to test whether the results we find here – both direct and strain mediated sensitivity to temperature– also holds for a larger number of strains and under more extreme temperature variation.

For pathogens the biotic environment may vary depending on the community of coinfecting pathogens sharing the same host. Coinfection is a common phenomenon across plant pathosystems (Tollenaere et al., 2016). In the *P. plantaginis* metapopulation, approximately half of the local pathogen populations support coinfection (Susi et al., 2015a). Theoretically coinfection has been proposed to change pathogen growth rates as strains compete for the same limited resources of the host, and there is some empirical support for this (Clement et al., 2012; Tollenaere et al., 2016; Suffert et al., 2018). Although previous studies on *P. plantaginis* have revealed coinfection to change both within- and between host dynamics (Susi et al., 2015a,b), here coinfection only had a significant effect on the number of chasmothecia produced. Higher production of resting spores under coinfection is expected to have profound fitness consequences as local pathogen populations go through a severe decline each winter (Tack and Laine, 2014), often resulting in local extinction (Jousimo et al., 2014). As coinfection varies spatially (Susi et al., 2015a), it can be a powerful mechanism maintaining diversity in this natural pathosystem.

Interestingly, we also did not detect a significant coinfection-by-temperature interaction for any of the measured life-history traits. It is likely that the effect of temperature in our experiment was so strong, that most of variation in these data is explained by the temperature gradient. However, it should also be noted that previous studies have shown strains to vary in how they respond to coinfection (Laine and Mäkinen, 2018). Hence, it is possible that the strains included in this study do not represent those that respond strongly to coinfection. Moreover, the effects of coinfection – either direct or mediated through an interaction with temperature – may not become apparent in the development of a single lesion but accumulate during the epidemic season (Susi et al., 2015a,b). Hence, a future study using more strains and allowing for several cycles of auto- and allo-infection would help elucidate whether coinfection changes infections outcomes also under pronounced abiotic variation.

## Conclusion

Here, we find both abiotic and biotic environments to impact pathogen life-history traits directly as well as through genotype specific responses. Moreover, we find that the effect of abiotic and biotic variation depends on pathogen life-history traits. Temperature variation affected all studied pathogen life-history traits and hence, we may expect it to be a powerful mechanism maintaining diversity during the epidemic season. We found coinfection to significantly affect the number of produced chasmothecia which is expected to have far-reaching consequences for pathogen survival from one epidemic season to the next (Tack and Laine, 2014). For an epiphytic pathogen such as *P. plantaginis*, it is not surprising to find high sensitivity to ambient temperature. Temperature fluctuations may be gradual (e.g., global warming), seasonal (e.g., summer vs. winter resource availability), or continuously fluctuating (e.g., daily temperatures). Our results suggest that *P. plantaginis* can cope with such variation in the environment, as we documented plastic responses in many life-history traits under temperature variation. We conclude that environmental variation is expected to be an important mechanism maintaining variation in pathogen populations, given that many studies have reported life-history trait plasticity in response to temperature variation (Blanford et al., 2003; Wolinska and King, 2009).

## Data Availability Statement

All data associated with this study is available on the Dryad Digital Repository: doi: 10.5061/dryad.fg25kk0.

## Author Contributions

EV and A-LL conceived and designed the experiments, analyzed the data, and wrote the paper. EV performed the experiments.

## Conflict of Interest Statement

The authors declare that the research was conducted in the absence of any commercial or financial relationships that could be construed as a potential conflict of interest.
