# Supplementary material for: Role of Temperature and Coinfection in Mediating Pathogen Life-History Traits
Source: Front Plant Sci. 2018 Nov 20;9:1670. doi: 10.3389/fpls.2018.01670 (PMC6256741; doi:10.3389/fpls.2018.01670)
Supplement: Supplementary file 1 [file Data_Sheet_1.pdf]

Supplementary Material

Role of temperature and coinfection in mediating pathogen life-history traits

Elise Vaumourin[1*], Anna-Liisa Laine[1]

[1] Research Centre for Ecological Change, University of Helsinki, Finland

*** Correspondence:**
Elise Vaumourin, Viikinkaari 1, PO Box 65, FI-00014 University of Helsinki, Finland. Phone: +33685919160, e-mail: elise.vaumourin@helsinki.fi

1 Supplementary Figures

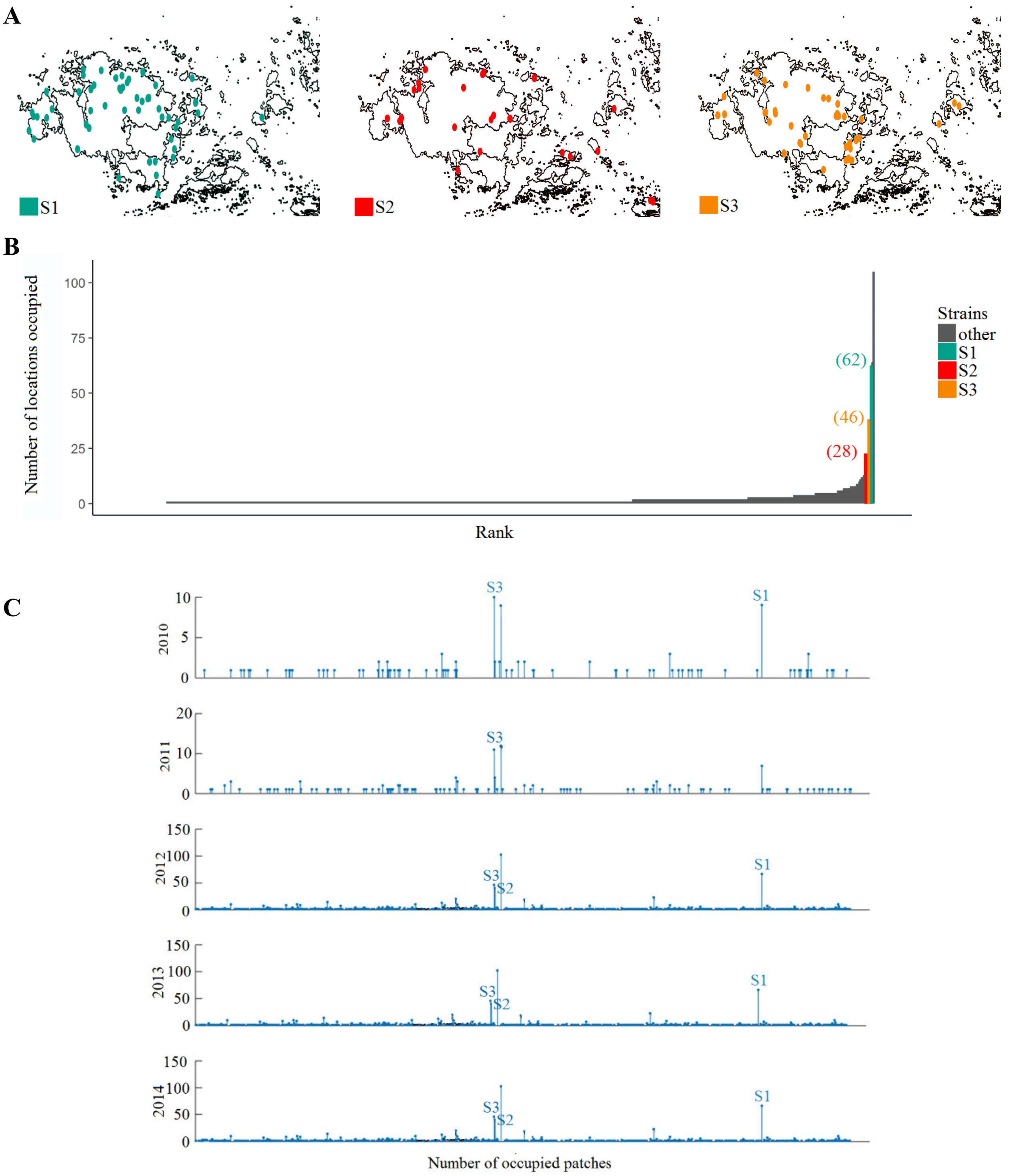

Supplementary Figure 1. Panel (A) displays the locations in which the studied strains (S1, S2 and S3) were found in 2015. Majority (395) of all the strains found that year were only found in one location. Panel (B) shows the frequency distribution of the number of occupied locations for all the strains found in 2015. The studied strains are shown in colors and the amounts of colonized locations for each strain are shown in parenthesis. Panel (C) shows the frequency distribution of the number of occupied locations for the studied strains (S1, S2 and S3) between 2010 and 2014.

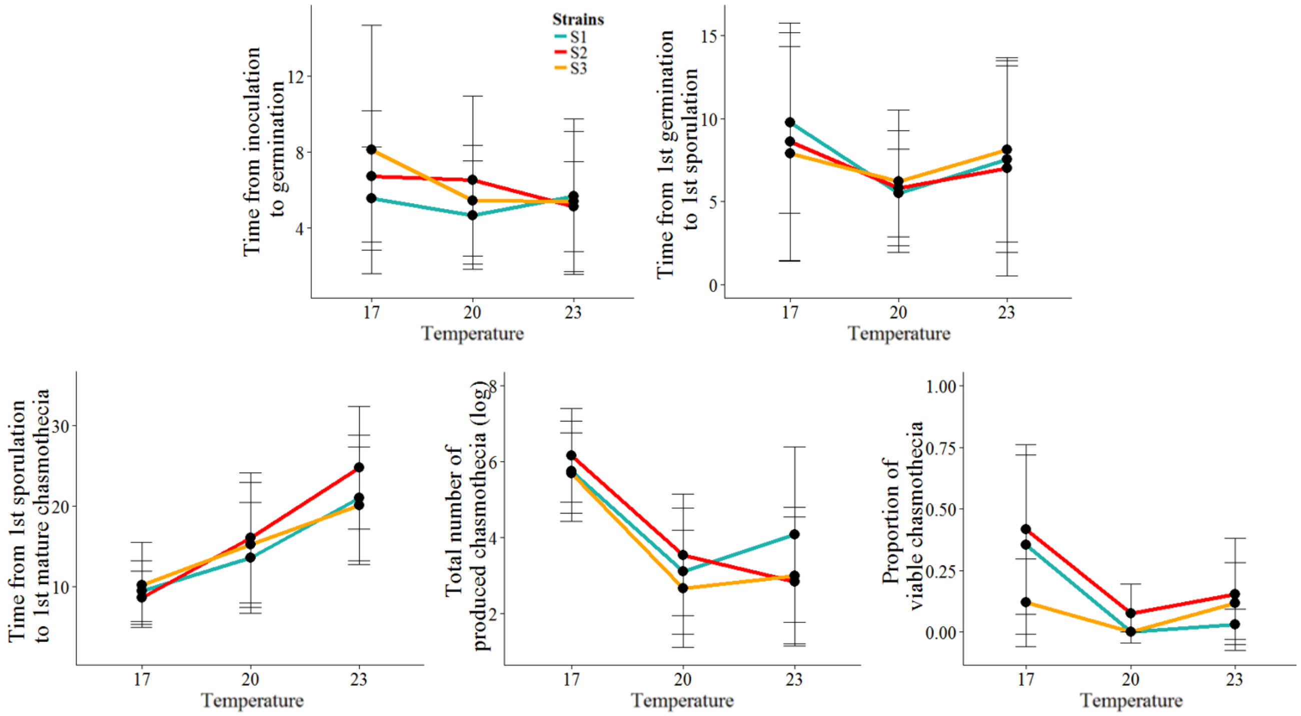

Supplementary Figure 2. The mean and standard deviation of the measured life-history traits of the three *Podosphaera plantaginis* strains in single infection treatment along a temperature gradient. The time is indicated in days.
